# Behavioral patterns in latrine use and handwashing in rural western Kenya: Age, time of day, and the role of perceived safety

**DOI:** 10.1371/journal.pone.0345954

**Published:** 2026-03-27

**Authors:** Noriko Tamari, Heidi E. Brown, Luigi Sedda, Gary L. Christopherson, Katherine D. Ellingson, Stephen Munga, Kacey C. Ernst

**Affiliations:** 1 Department of Epidemiology and Biostatistics, College of Public Health, University of Arizona, Tucson, Arizona, United States of America; 2 Lancaster Ecology and Epidemiology Group, Lancaster Medical School, Lancaster University, Lancaster, United Kingdom; 3 School of Geography and Development, University of Arizona, Tucson, Arizona, United States of America; 4 Kenya Medical Research Institute (KEMRI), Centre for Global Health and Research, Kisumu, Kenya; Bangladesh Institute of Social Research (BISR) Trust, BANGLADESH

## Abstract

Latrine use enhances health benefits, safety, dignity, and social status. Despite increased latrine coverage, some children and adults do not consistently use latrines. The present study aimed to describe latrine use and handwashing after urination and defecation by age and time of day, and to explore factors associated with latrine use at each time of day. A cross-sectional, population-based survey was conducted from July 17 to September 21, 2023 in western Kenya, targeting individuals aged 4 years or older (n = 528 analyzed). Overall, latrine use tended to be more frequent among adults than children, for defecation than urination, and during the daytime and early morning compared with at night. Handwashing practices after urination and defecation showed similar patterns. For urination, compared with young adults (18–39 years), young children (4–10 years) were less likely to use latrines across all times of day, with reductions of approximately 60–85%. For defecation, compared with adults (18 + years), young children were even less likely to use latrines across all times of day (approximately 90–95% lower likelihood). Similarly, adolescents (11–17 years) had approximately 75% lower latrine use for defecation at night and early in the morning compared with adults. In contrast, individuals who felt safe walking to the latrine at night were substantially more likely to use latrines for both urination and defecation than those who perceived the walk as neither safe nor unsafe or unsafe. Therefore, simple, low-cost interventions, such as promoting the use of flashlights, constructing latrines closer to households, and better connecting sanitation knowledge to daily practices, are crucial for improving sanitation behaviors.

## Introduction

Latrine use enhances health benefits, safety, dignity, and social status [1–3]. Conversely, the practice of open defecation, such as defecating in the bush, fields, ditch or bodies of water, leads to the spread of diseases including cholera, dysentery, hepatitis, typhoid, intestinal worm infections, polio, trachoma or schistosomiasis [4–6]. Repeated exposure to these diseases can adversely affect the physiological well-being of women [7] and impair the growth of children [8,9]. Eliminating open defecation is therefore crucial for improving physical and mental health. Accordingly, Sustainable Development Goal (SDG) target 6.2 aims to achieve access to adequate and equitable sanitation and hygiene for all and to end open defecation by 2030 [10]. Evidence on health impacts of water, sanitation and hygiene (WASH) interventions has been mixed [11–19]. Recent analyses suggest that the reductions in diarrheal disease have historically been achieved through sewerage systems and broader environmental improvements, rather than basic sanitation [16]. The limited effectiveness of basic sanitation interventions may be explained, in part, by the gap between latrine ownership and actual use, as open defecation persists even when latrines are available within the households [20–26].

According to the Kenya Malaria Indicator Survey in 2020, household sanitation facilities were predominantly improved latrines (e.g., ventilated improved pit, VIP, and pit latrines with slabs) (66%), followed by unimproved facilities (e.g., pit latrine) (28%) and open fields (6%) [27]. Despite an overall latrine coverage of 94%, only 89% of adults and 66% of children consistently used a latrine [23]. Previous studies have shown that open defecation is associated with lower income or poor sanitary conditions [20,22,24–26], indicating that unimproved facilities may deter consistent latrine use. Although improvements to latrine infrastructure can be costly and unaffordable for some households [23,28], high community-level latrine coverage and the presence of neighboring improved latrines have been associated with health benefits [29,30]. These findings highlight the importance of community-wide latrine use regardless of whether the facilities are improved or unimproved.

Barriers to latrine use and handwashing practices have been well documented, including poor latrine conditions, lower income, education, larger family size, and perceived unsafe access to latrines [26,31–33]; however, most studies have focused on the behaviors of school children, household heads, or women and primarily on defecation during daytime hours [7,20,24–26,34]. Consequently, little is known about the behaviors by age, time of day, and type of latrine use (defecation vs. urination). For example, elderly individuals may contribute to environmental contamination through practicing open defecation due to long-standing habits or cultural norms [35–37]. Open urination also poses risks for parasitic and diarrheal diseases [38,39], and some individuals may be discouraged from using outdoor latrines at night or in the morning due to security concerns and latrine overcrowding [23,40–43]. Handwashing practices can further influence disease transmission. Individuals who use latrines but do not wash their hands increase the risk of contaminating food and drinking water and transmitting pathogens through direct contact [44,45]. Therefore, a comprehensive understanding of latrine use and handwashing behaviors for urination and defecation across age groups and times of day is essential.

The present study aimed to 1) describe latrine use for urination and defecation among children and adults at three different time points—the daytime, at night, and early morning; 2) describe handwashing station availability and handwashing practices after urination and defecation at each time point and before eating; and 3) explore factors associated with latrine use for urination and defecation at each time point. We hypothesized that latrine use would be more common in the improved latrine structure than in the unimproved ones, in the absence of feces around latrines rather than in their presence, at shorter distances to latrines than at longer distances, among younger adults compared with children and the elderly, for defecation rather than urination, and during the daytime rather than at night or early morning. Understanding these behaviors will provide insights for the development of practical strategies to improve latrine use among households with a latrine.

## Methods

### Study area

The study was conducted in western Kenya within Central Kabar sublocation (6.2 km^2^) of Miwani, 30 km east of Kisumu, Kenya. The altitude is approximately 1,200 m above sea level. The rainfall pattern is bimodal, with a long rainy season from April through June and a short rainy season from November to December. *Plasmodium falciparum* parasite infection (malaria) occurs year-round with seasonal increases [46]. Most of the population are Luo ethnicity and are subsistence farmers, with some of them employed by local commercial sugarcane and rice growers. A total of 631 residential compounds were enumerated in the study area, comprising 1,018 households with a total of 3,898 residents. Only 3% (n = 19) of compounds had at least one household with an indoor latrine. The majority (63%, n = 400) had outdoor latrines, and only 3% (n = 15) of them had more than one latrine.

### Study design

A cross-sectional population-based household survey was conducted from July 17 to September 21, 2023.

### Populations

Our target population included individuals aged 4 years or older, who potentially use outdoor latrines [47] and reside in households meeting our criteria. *Household definition:* In the study area, some residential compounds consist of multiple house structures (e.g., a son’s family house and a second wife’s house) and outdoor latrines are shared among individuals living in multiple households within the compound. We define a household as individuals who live together and share meals in the same structure, but not necessarily sleep together. Some children sleep in the second wife’s house (their relative’s house) or the main house (their grandparents’ house) within the same compound. If at least one outdoor latrine is present in a compound, all households in the compound are considered to have outdoor latrines. *Inclusion criteria*: Households that had at least one child aged 4–17 years, a single typical outdoor latrine (i.e., iron/wood door, brick/iron/mud wall, iron roof with open eaves) in the compound, and a typical house structure (i.e., mud wall, iron roof with open eaves). *Exclusion criteria*: Households belonging to a compound with at least one household having an indoor latrine or multiple outdoor latrines. The latrine criteria were used to identify the latrine used by each individual and to account for latrine-related characteristics (i.e., when multiple latrines were present within a compound, individuals might use them interchangeably, making it challenging to assess the effects of specific latrine characteristics). *Sampling*: Households meeting the eligibility criteria were randomly selected from the enumerated list of households and their respective compounds within the study site. The sample size was calculated at the individual level, while recruitment was conducted at household level due to challenges in recruiting only certain members within a household. Therefore, all residents of age of 4 years or older in the selected households were included in the study. The inclusion and exclusion criteria were applied to households during the enumeration phase because the study area frequently experiences population movement related to school and work, as well as modifications or refurbishment of latrine structures. Consequently, households were enrolled even if family composition or latrine characteristics had changed between enumeration and recruitment. These participants were retained to assess latrine use dynamics in a study area characterized by frequent population movement and structural modifications, reflecting real-world conditions.

### Data collection

#### Training.

We provided field personnel with training in research ethics and survey administration. We used tablets for data collection, directly entering the data into REDCap (Research Electronic Data Capture).

#### Mapping and enumeration.

Prior to the present study, the study site was enumerated, and field personnel directly observed the houses and outdoor latrines. Additionally, they interviewed the household heads regarding the number of indoor latrines and the number of infants (0–3 years), children (4–10 years), adolescents (11–17 years), and adults (18 years or older) as part of a household survey. Geographical coordinates of house, outdoor latrine and kitchen structures were recorded using a handheld global positioning system (GPS) (Garmin, Olathe, KS, USA).

#### Household survey.

Field personnel visited selected households to interview the household heads and collect the additional information. They also directly observed the latrine structures to confirm any changes since the study site was mapped and enumerated.

#### Individual survey.

Field personnel conducted structured standardized interviews on latrine use and handwashing practices with individuals who consented to participate in the present study. Children aged 8–17 years were interviewed following assent and parental permission. Caretaker permission was obtained for children aged 4–7 years. The female caretaker was interviewed on behalf of these children when they were not able to understand and respond to the interviewer’s questions. Field personnel interviewed participants about latrine use behaviors during the daytime, at night and in the early morning. We defined “latrine use at night” as “using latrines when it was dark,” which was normally between 7 pm and 6 am, and “latrine use in the early morning” as “using latrines soon after getting up”, which was normally between 6 am and 7 am.

### Sample size determination

The sample size was calculated to obtain a representative population of latrine users. Open Epi Version 3 statistical software was used for a single population proportion formula [48,49], considering a population size of 433 for children and 391 for adults, a design effect of 1, and the following assumptions of latrine use: 66% for children and 89% for adults [23]. With a margin of error of 5% at the 95% confidence level, the required sample size was 193 for children and 109 for adults. After accounting for 5% of absent or incomplete data, the minimum required sample sizes were 204 children and 115 adults from 159 households. Given the different aims of the study, we recruited 265 children and 263 adults based on the sample size calculation of a study about WASH and *P. falciparum* infection. These sample sizes were sufficient to analyze in the present study.

### Study variables

#### Latrine use behaviors and handwashing practice.

Latrine use behavior included latrine use for each of urination and defecation during the daytime, at night, and early morning. In analytical models, each latrine use behavior was categorized as ‘latrine use’ with ‘other’ (bucket or open fields) as a reference. Handwashing practice information for each of urination and defecation during the daytime, at night, and early morning, as well as before eating, was recorded.

#### Other variables.

*Individual level variables*: Variables at individual level included sex (female/male), age, respondent type (self/caretaker), sleeping place (own house/kitchen/other structure within the compound), perceived safety walking to a latrine at night (safe/neither/unsafe), and safety concerns related to walking to or using a latrine at night (none/age/animal/darkness/fear of robbery/poor latrine conditions/other safety concerns).

*Household level variables*: Variables at household level were number of residents in total (i.e., residents under 4 years) and number of targeted residents (i.e., residents aged 4 or older), socioeconomic status (SES) (low/middle/high) of the household, education level of the caretaker (none/some primary/completed primary/completed secondary), the presence of a bucket in the house (yes/no), and distance from a living house to a latrine (in meters) [47,50,51].

*Compound level variables*: Variables regarding outdoor latrines included construction year of a latrine (≤5 years/6–10 years/ ≥ 11 years), funder for the latrine construction (individual household/joint community/NGO/other), presence of handwashing station (yes/no), cleaning frequency of latrine (daily/weekly/biweekly/monthly/rarely), materials used for cleaning (ash/detergent/sweep or mopping), feces on floor of latrine (no visible feces/small amount of visible feces/very visible feces), type of a latrine (pit/VIP), presence of bath space attached to a latrine (yes/no), materials used for door (iron/wood/polyethene or rag/no door), floor (cement or tile/mud/wood or plastic), wall (brick or cement/iron/mud/polythene) and roof (iron), eaves status (open), and potential number of people using a latrine (i.e., people aged at 4 years or older) [47,50,51].

*Recorded variables*: Age was calculated from the date of birth, and categorized as 4–10 years (young children), 11–17 years (adolescent), 18–39 years (young adult), 40–59 years (middle adult), and 60 years or older (elderly) to understand the behaviors of each generation. Some variables were collapsed into fewer categories because these were too few responses. In the analyses of defecation behavior, the adult age group was regrouped into a single category due to lower variability. SES for each household was assessed using a composite household material wealth index. The index was based on possession of consumer goods (owning radio, TV, tablet, laptop, smartphone, bicycle, motorcycle, and car), source of drinking water, main source of electric lighting and employment presence. We recorded the presence or absence of each item, and assigned a numerical score to each household through multiple correspondence analysis (MCA) [52–54]. These scores were then divided into tertiles to obtain a proxy of socioeconomic “classes” (i.e., low, middle and high). The distances from individuals’ sleeping places to the latrine and from households to the latrine were calculated using the GPS coordinates. The GPS devices (Garmin GPSMAP®64s) used are accurate to within 3.65 meters [55].

### Data analyses

*Descriptive statistics*: Summary tables were made for data regarding demographics and behavioral factors at the individual levels, social factors at the household level, and latrines at the compound level (note: latrines were built at the compound level). Proportions were computed for categorical variables. The mean, median, range and standard deviation (SD) were computed for numeric variables. Figures were created for latrine use and handwashing practices for each age group. In addition, proportions and 95% confidence intervals (CI) were calculated for consistent latrine use for urination, defecation, and both throughout the day by each age group.

*Factors associated with latrine use for urination and defecation*: A Generalized Variance Inflation Factor (GVIF) was used to assess collinearity among the covariates, indicating no covariates had a GVIF greater than three [56]. Since participants were recruited from the same households, households were considered as a random effect in generalized linear mixed models (GLMMs). However, the GLMMs and generalized estimating equations (GEE) did not converge, and therefore data were fitted using log-binomial regression models in Integrated Nested Laplace Approximation (R-INLA), with households as a random effect [57]. The prevalence ratio (PR) was used to explore factors associated with outdoor latrine use for each of urination and defecation at three time points: daytime, nighttime and early morning. We constructed six models for each of urination and defecation at three time points.

*Sensitivity analysis*: A sensitivity analysis was conducted to confirm the robustness of the findings. Because the criteria were applied to households during the enumeration period, households were enrolled even if their family composition or latrine structures had changed between enumeration and recruitment. As a result, the criteria were not necessarily met at the time of recruitment. In the sensitivity analysis, we used a dataset that excluded individuals from households that did not meet inclusion criteria at the recruitment period. All data were analyzed using R (version 4.4.1) [58].

### Informed consent

Prior to data collection, the field personnel visited households and explained the goals, risks, benefits, and voluntary participation in the study. Written consent was obtained from individuals aged 18 or older years and parental consent and child assent from children aged 7–17 years. Parental consent was obtained from children aged 4–6 years.

### Ethical statement

The present study was approved by the KEMRI-SERU (SERU4736) and the University of Arizona ethical review boards with deferral of primary oversight from the University of Arizona to KEMRI.

## Results

### Sample

**Fig 1:**
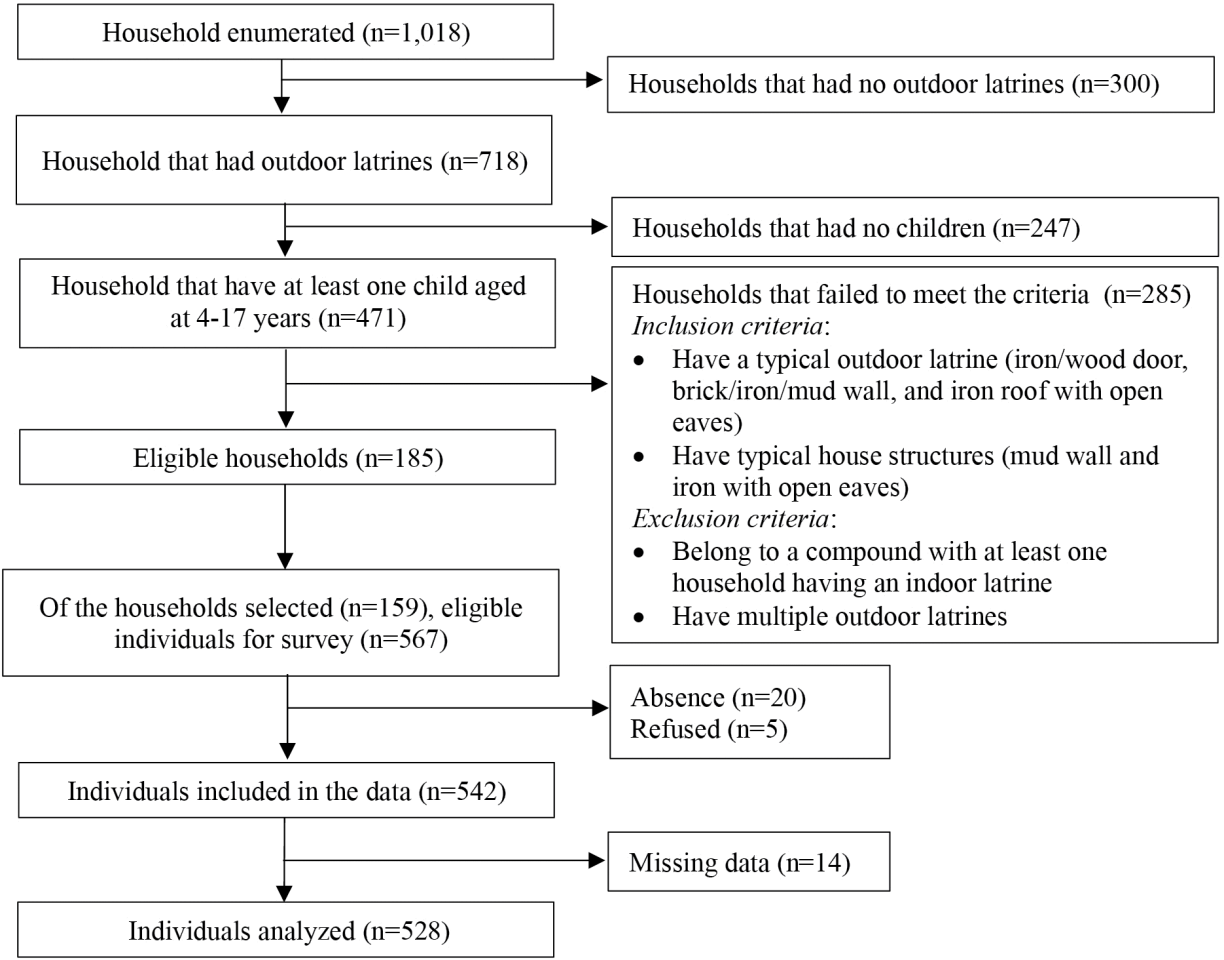
Flowchart showing selection of participants and households. Note: Some compounds consist of multiple house structures and outdoor latrines are shared among individuals living in these houses within the compound. A household is defined as individuals who live together and share meals in the same structure. If outdoor latrines are present in a compound, all households in the compound are considered to have outdoor latrines. Of 159 households within 126 compounds analyzed, multiple households were selected from 24 compounds.

A total of 185 households were eligible for the present study, and 159 households were randomly selected. Among the 567 eligible individuals in the selected households, 542 individuals participated in the present study, while five declined participation and 20 were absent. We excluded 14 individuals from the dataset due to missing data. The final sample size included 528 individuals from 159 households within 126 compounds (Fig 1).

Of the 528 individuals for whom complete data was available, 57% were female, and the mean age was 22 years (SD = 18). The age distribution was as follows: 26% were aged 4–10 years (young children), 24% were aged 11–17 years (adolescents), 28% were aged 18–39 years (young adults), 16% were aged 40–59 years (middle-aged adults), and 6% were aged 60 years or older (elderly) (Table 1).

**Table 1:** Individual characteristics and perceived safety related to latrine use (n = 528).

	Individuals					
Variable	Age, years					
	4-10	11-17	18-39	40-59	60+	Total
***Sex***, n (%)						
Male	63 (46.7)	59 (46.1)	52 (35.6)	37 (43.0)	15 (45.5)	226 (42.8)
Female	72 (53.3)	69 (53.9)	94 (64.4)	49 (57.0)	18 (54.5)	302 (57.2)
***Respondent type***, n (%)						
Self	59 (43.7)	122 (95.3)	146 (100)	86 (100)	33 (100)	446 (84.5)
Caretaker	76 (56.3)	6 (4.7)	0	0	0	82 (15.5)
***Sleeping place***^a^, n (%)						
Other structure within the compound	8 (5.9)	11 (8.6)	5 (3.4)	0	0	24 (4.5)
Kitchen	24 (17.8)	54 (42.2)	37 (25.3)	13 (15.1)	4 (12.1)	132 (25.0)
Own house	103 (76.3)	63 (49.2)	104 (71.2)	73 (84.9)	29 (87.9)	372 (70.5)
***Perceived safety walking to a latrine at night***, n (%)						
Unsafe	81 (60.0)	26 (20.3)	17 (11.6)	4 (4.7)	2 (6.1)	130 (24.6)
Neither	2 (1.5)	1 (0.8)	2 (1.4)	0	0	5 (0.9)
Safe	52 (38.5)	101 (78.9)	127 (87.0)	82 (95.3)	31 (93.9)	393 (74.4)
***Distance from a sleeping place to a latrine***, m						
Mean (SD)	27.6 (12.4)	28.9 (13.0)	30.4 (13.3)	29.3 (12.7)	26.6 (9.6)	28.9 (12.7)
Median [range]	26.0 [2.0,62.0]	27.5 [6.0,90.0]	30.0 [2.0,62.0]	29.0 [3.0,60.0]	26.0 [10.0,46.0]	28.0 [2.0,90.0]
***Safety concerns walking to or using a latrine at night***^b^, n (%)						
None	78 (57.8)	112 (87.5)	137 (93.8)	84 (97.7)	29 (87.9)	440 (83.3)
Age (young or old)	23 (17.0)	1 (0.8)	2 (1.4)	0	0	26 (4.9)
Animal (e.g., dogs, snakes)	12 (8.9)	5 (3.9)	1 (0.7)	0	0	18 (3.4)
Darkness	9 (6.7)	0	1 (0.7)	1 (1.2)	0	11 (2.1)
Fear of robbery	6 (4.4)	3 (2.3)	1 (0.7)	0	1 (3.0)	11 (2.1)
Poor latrine conditions	7 (5.2)	4 (3.1)	4 (2.7)	1 (1.2)	3 (9.1)	19 (3.6)
Other safety concerns	0	3 (2.3)	0	0	0	3 (0.6)
***Total***, n (%)	135 (25.6)	128 (24.2)	146 (27.7)	86 (16.3)	33 (6.3)	528 (100)

m, meter; n, number; SD, standard deviation.

^a^In the study area, some residential compounds consist of multiple house structures. In addition to the main house, these may include a son’s family house and a second wife’s house. Some children sleep in one of these houses within the compound.

^b^Self-perception among children aged 4–10: none (n = 40), age (n = 0), animal (n = 7), darkness (n = 5), fear of robbery (n = 5), and poor latrine conditions (n = 2); among children 11–17: none (n = 107), age (n = 1), animal (n = 5), fear of robbery (n = 2), poor latrine conditions (n = 4), and other safety concerns (n = 3).

Individuals primarily slept in their own house (71%) or kitchen structures (25%), while a small proportion slept in other house structures (e.g., relative’s or grandparents’ house) within the compound (5%). The mean distance from a sleeping place to a latrine was 29 m (SD = 13). The perception of walking to a latrine as safe at night was reported by 39% for young children, which was about half of the proportion reported by adolescents (79%). Young adults had a lower perception of safety (87%) compared with middle-aged (95%) and senior adults (94%). Regarding safety concerns related to walking to or using a latrine at night, young children reported that they were too young to use latrines at night (17%), fear of animals (9%), darkness (7%), robbery (4%), and poor latrine conditions (5%). A few individuals in other age groups also reported fear of poor latrine conditions (Table 1).

The mean number of eligible residents per household (i.e., 4 years or older) was 4 (SD = 2). Education level of household heads was 4% for non-education, 25% for some primary education, 43% for completed primary education, and 28% for completed secondary education or higher (Table 2). The proportion of households with a bucket inside the house was 20%, and the mean distance from a house to a latrine was 30 meters (m) (SD = 13) (Table 2).

**Table 2:** Household sociodemographic characteristics and sanitation-related factors (n = 159).

Variable	Household
***Num of residents in total***	
Mean (SD)	4.0 (2.1)
Median [range]	4 [1,11]
***Num of residents targeted*** ^a^	
Mean (SD)	3.5 (1.9)
Median [range]	3 [1,11]
***SES***, n (%)	
Low	54 (34.0)
Middle	52 (32.7)
High	53 (33.3)
***Education level of caretaker***, n (%)	
None	6 (3.8)
Some primary	40 (25.2)
Completed primary	68 (42.8)
Completed secondary	34 (21.4)
Completed tertiary	11 (6.9)
***Have a bucket in a house***, n (%)	
No	127 (79.9)
Yes	32 (20.1)
***Distance from a house to a latrine***, m	
Mean (SD)	30.0 (12.7)
Median [range]	29.8 [2.2,61.7]

m, meter; min, minute; n, number; num, number; SD, standard deviation; SES, socio economic status; VIP, ventilated improved pit.

^a^Number of residents aged 4 years or older.

### Characteristics of outdoor latrines surveyed

Primary types of latrines observed were pit latrines (87%) or VIP (13%). The most common construction material was iron door (83%), mud floor (58%) and iron wall (52%). The mean number of individuals potentially using latrine (i.e., number of residents aged 4 years or older in a compound) was 6 (SD = 4) (Table 3). Of 126 latrines selected based on the enumerated list, 17 had changes in structural material (Table 3). Additionally, no children lived in 51 households due to absence or migration (Table 2). In total, 56 households experienced changes in latrine structures or family composition between the enumeration and selection phases. Most latrines were privately funded (91%), and 74% had been constructed within the past five years. Only 3% had a handwashing station. Cleaning routines were primarily conducted on a daily (38%) or weekly (44%) basis with sweep or mopping, ash, or detergent. Over 75% of latrines showed no presence of feces on the floor (Table 3).

**Table 3:** Characteristics of outdoor latrines at the compound level (n = 126).

Variable	n (%)
***Built year*** ^a^	
2019–2023 (≤5 years)	93 (74.4)
2015–2018 (6–10 years)	23 (18.4)
≤ 2014 (≥11 years)	9 (7.2)
***Funder***	
Individual household	114 (90.5)
Joint community	1 (0.8)
NGO	3 (2.4)
Other	8 (6.3)
***Handwashing station***	
No	122 (96.8)
Yes	4 (3.2)
***Frequency of Cleaning***	
Daily	48 (38.1)
Weekly	55 (43.7)
Biweekly	11 (8.7)
Monthly	2 (1.6)
Rarely	10 (7.9)
***Use of Cleaning material***	
No	3 (2.4)
Yes	123(97.6)
***Cleaning material used*** ^b^	
*Use ash (n = 123)*	74 (58.7)
*Use detergent (n = 123)*	50 (40.7)
*Sweep/mopping (n = 123)*	93 (75.6)
***Feces on floor of latrine***	
No visible feces	97 (77.0)
Small amount of visible feces	28 (22.2)
Very visible feces	1 (0.8)
***Type***	
Pit	110 (87.3)
VIP	16 (12.7)
***Bath space is attached***	
No	108 (85.7)
Yes	18 (14.3)
***Door***	
Iron	104 (82.5)
Wood	6 (4.8)
Polythene/Rag	13 (10.3)
No door	3 (2.4)
***Floor***	
Cement/Tile	51 (40.5)
Mud	73 (57.9)
Wood/Plastic	2 (1.6)
***Wall***	
Brick/Cement	19 (15.1)
Iron	66 (52.4)
Mud	40 (31.7)
Polythene	1 (0.8)
***Roof***	
Iron	126 (100)
***Eaves***	
Open	126 (100)
***Potential number of people using a latrine*** ^c^	
Mean (SD)	6.1 (3.9)
Median [range]	5 [1,26]

n, number; NGO, non-governmental organization; SD, standard deviation; VIP, ventilated improved pit.

^a^n = 1 was unknown.

^b^Interviewed those who responded to use of cleaning material (n = 123).

^c^Number of residents aged 4 years or older in the compound.

### Latrine use behaviors

**Fig 2:**
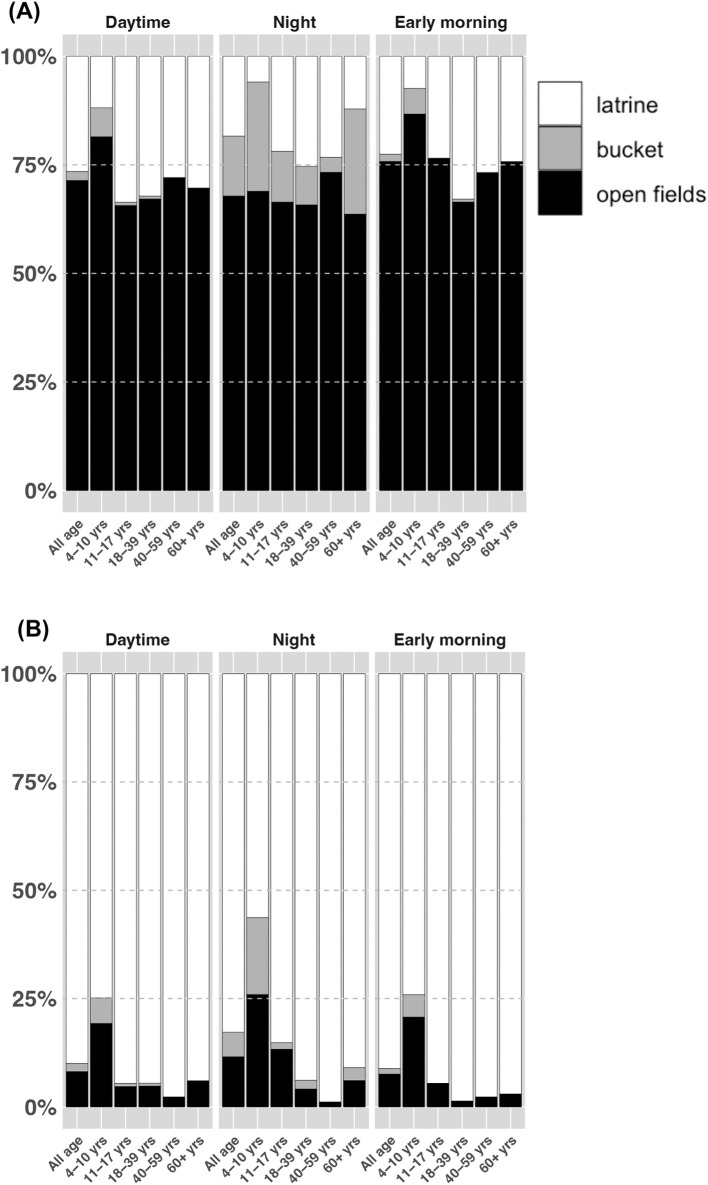
Latrine use during the day time, at night, and in the early morning by age. **(A)** After urination. **(B)** After defecation.

Latrine use for urination was 27% during the daytime, 18% at night, and 23% in the early morning, whereas latrine use for defecation was 90% during the daytime, 83% at night, and 91% in the early morning (Fig 2 and S1 Table). Additionally, around 25% of individuals in these youngest and elderly groups used buckets for urination at night, compared with 4–12% in other age groups (Fig 2 and S1 Table).

Consistent use of latrine for urination throughout the day was 12% (95%CI: 8.3, 16.5) for children and 19% (95%CI: 14.8, 24.6) for adults. Similarly, consistent use for defecation was 66% (95%CI: 59.7, 71.4) for children and 91% (95%CI: 86.2, 93.7) for adults (Table 4).

**Table 4:** Consistent latrine use for urination and defecation during the daytime, at night, and in the early morning, by age group.

Variable	Age, years,% (95%CI)					Children	Adults	
	4-10	11-17	18-39	40-59	60+	4-17	18+	All
***Urination***								
Other^a^	94.8 (89.2,97.7)	81.3 (73.2,87.4)	76.7 (68.9,83.1)	84.9 (75.2,91.4)	87.9 (75.2,91.4)	88.2 (83.5,91.7)	80.8 (75.4,85.3)	84.5 (82.0,87.4)
Always use	5.2 (2.3,10.8)	18.8 (12.6,26.8)	23.3 (16.9,31.1)	15.1 (8.6,24.8)	12.1 (8.6,24.8)	11.8 (8.3,16.5)	19.2 (14.8,24.6)	15.5 (12.6,19.0)
***Defecation***								
Other^a^	48.1 (39.5,56.9)	19.5 (13.3,27.7)	11.0 (6.6,17.5)	4.7 (1.5,12.1)	15.2 (5.7,32.7)	34.2 (28.6,40.3)	9.4 (6.3,13.8)	21.8 (18.4,25.6)
Always use	51.9 (43.1,60.5)	80.5 (72.3,86.7)	89.0 (82.5,93.4)	95.3 (87.9,98.5)	84.8 (67.3,94.3)	65.8 (59.7,71.4)	90.6 (86.2,93.7)	78.2 (74.4,81.6)
***Urination and Defecation***								
Other^a^	94.8 (89.2,97.7)	82.3 (74.0,88.0)	77.4 (69.6,83.7)	84.9 (75.2,91.4)	87.9 (70.9,96.0)	88.6 (84.0,92.1)	81.1 (75.8,85.6)	84.8 (81.4,87.7)
Always use	5.2 (2.3,10.8)	18.0 (12.0,26.0)	22.6 (16.3,30.4)	15.1 (8.6,24.8)	12.1 (4.0,29.1)	11.4 (7.9,16.3)	18.9 (14.4.24.2)	15.2 (12.3,18.6)

^a^Other included those who reported using open field at least once during the daytime, at night, or early morning.

CI, confidence interval.

### Handwashing practices

**Fig 3:**
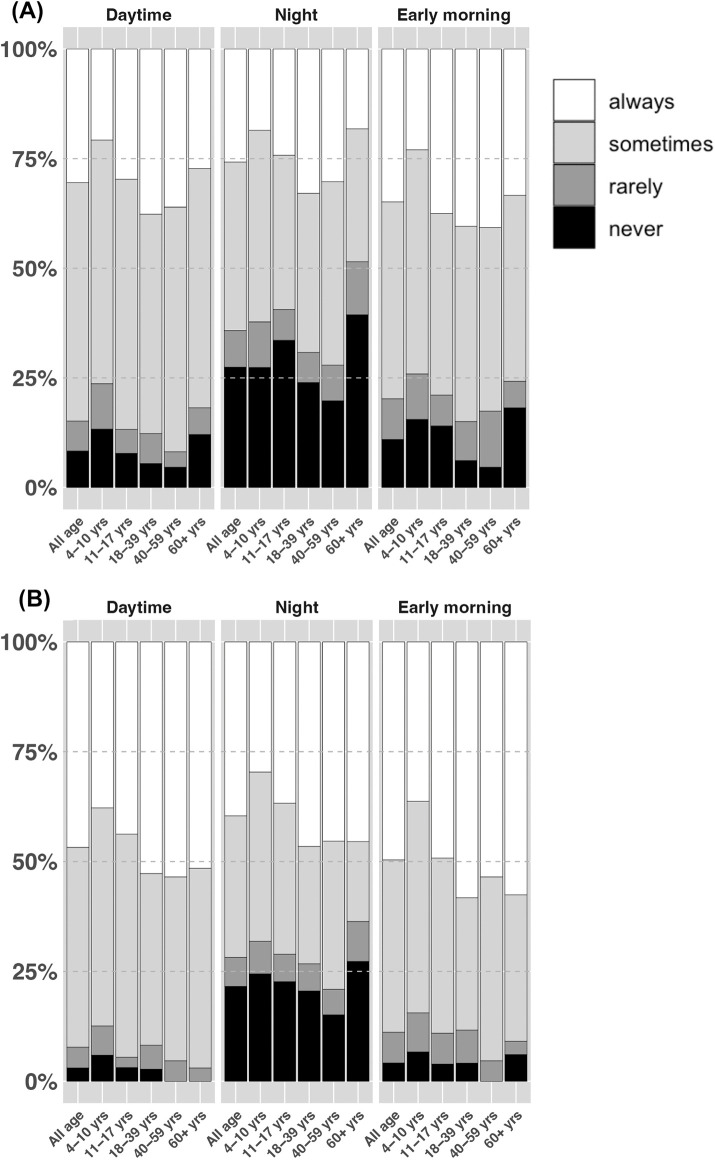
Handwashing practice during the day time, at night, in the early morning by age. **(A)** After urination. **(B)** After defecation.

Nearly all individuals (99%) practiced handwashing before eating. However, handwashing practices after using latrines varied by age, the type of latrine use, and time of using. Always handwashing after urination was reported by 21% to 30% of children and 30% to 40% of adults, while handwashing after defecation was reported by 33% to 43% of children and 46% to 57% of adults. Overall, handwashing was most frequently practiced in the early morning, followed by daytime and night. Notably, the elderly always practiced handwashing after urination (18% to 33%) at similar proportions to children. Approximately 4% of participants never practiced handwashing after defecation during the daytime or early morning but this number increased to 22% at night (Fig 3 and S2 Table).

### Factors associated with latrine use for urination

Unadjusted and adjusted effects from regression analyses demonstrated similar associations for use of latrines for urination and defecation at different time points. Variables that were only associated in unadjusted regression included the number of individuals potentially using latrines during the day (PR: 0.95, 95%CrI: 0.90, 0.99) and the absence of feces around latrines compared with presence of feces, which was significant during the day (PR:1.89, 95%CrI: 1.15, 3.09) and early morning (PR: 1.77, 95%CrI: 1.05, 2.97). Significance was observed only in the adjusted regression for having mud floor in latrines compared with a cement/tiles floor, which was associated with increased use of latrines for urination during the day (aPR: 1.79, 95%CrI: 1.07, 2.99) and early morning (aPR: 2.22, 95%CrI: 1.26, 3.88) (S3 Table).

**Fig 4:**
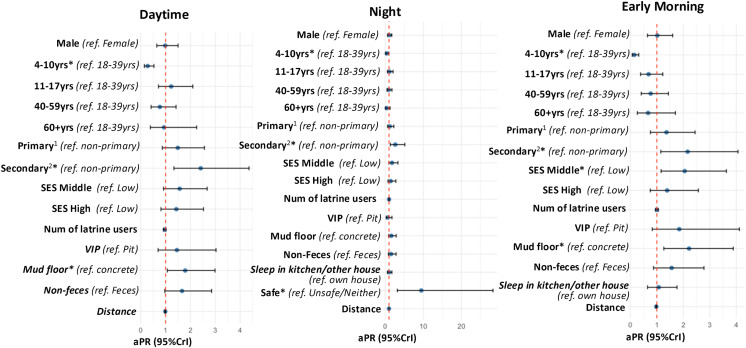
Unadjusted and adjusted analyses of factors associated with latrine use for urination during the daytime, at night, and in the early morning (n = 528). aPR, adjusted prevalence ratio; CrI, credible interval; num, number; *ref*, reference; SES, socio economic status; VIP, ventilated improved pit; yrs, years. *Credible evidence; ^1^Completed primary education; ^2^Completed secondary education.

Adjusted regression analyses showed that, compared with the young adult age group (18–39 years), the youngest age group (4–10 years) was 72% less likely to use latrine for urination during the daytime (aPR:0.28, 95%CrI: 0.15, 0.53), 59% less at night (aPR: 0.41, 95%CrI: 0.17, 0.98), and 85% less early in the morning (aPR: 0.15, 95%CrI: 0.07, 0.32). Having a caretaker who had completed secondary education was associated with an increase in latrine use for urination by 2.41 times (95%CrI: 1.34, 4.36) during the daytime, 2.64 times (95%CrI: 1.35, 5.15) at night, and 2.16 times (95%CrI: 1.15, 4.06) early in the morning compared with having a caretaker with incomplete primary education. Individuals from middle SES households were 2.05 times (95%CrI 1.16, 3.62) more likely to use latrines early in the morning, compared with those from low SES households. Additionally, individuals who felt safe walking to the latrine at night were 9.47 times (95%CrI: 3.17, 28.28) more likely to use latrines for urination at night compared with those who perceived the walk as neither safe nor unsafe or unsafe (S3 Table and Fig 4).

### Factors associated with latrine use for defecation

For latrine use for defecation, unadjusted and adjusted effects from regression analyses showed similar tendencies. The only significant unadjusted associations for the prevalence of latrine use for defecation were sex, with lower use among women than men (PR: 0.56, 95%CrI: 0.35, 0.91), and number of individuals potentially using the same latrine (PR: 0.95, 95%CrI: 0.91, 0.99), with an increase in the number of users associated with lower latrine use. In contrast, significance was observed only in the adjusted regression for adolescents compared with adults, where prevalence of latrine use for defecation early in the morning was lower among adolescents (aPR: 0.25, 95%CrI: 0.08, 0.84) (S4 Table).

**Fig 5:**
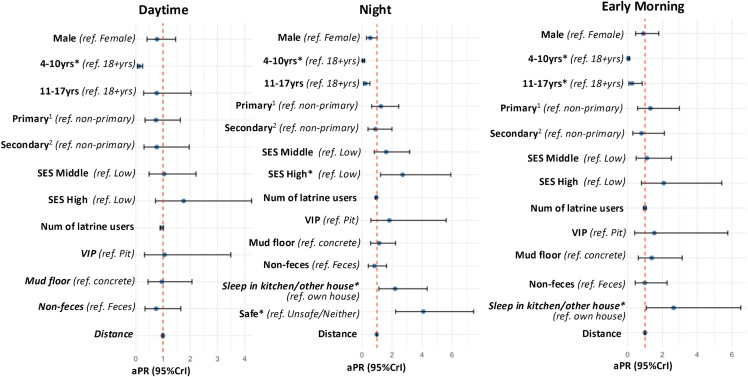
Unadjusted and adjusted analyses of factors associated with latrine use for defecation during the daytime, at night, and in the early morning (n = 528). aPR, adjusted prevalence ratio; CrI, credible interval; num, number; *ref*, reference; SES, socio economic status; VIP, ventilated improved pit; yrs, years. *Credible evidence; ^1^Completed primary education; ^2^Completed secondary education.

Adjusted regression analyses showed that, compared with adults, young children (4–10 years) were 88% less likely to use latrines for defecation during the daytime (aPR: 0.12, 95%CrI: 0.06, 0.25), 92% less likely at night (aPR: 0.08, 95%CrI: 0.04, 0.18), and 96% less likely early in the morning (aPR: 0.04, 95%CrI: 0.02, 0.11). Similarly, adolescents were 77% less likely to use latrines for defecation at night (aPR: 0.23, 95%CrI: 0.10, 0.53) and 75% less likely early in the morning (aPR: 0.25, 95%CrI: 0.08, 0.84). Sleeping in kitchen or other structures was associated with an increased prevalence of latrine use for defecation, with an increase of 2.21times at night (95%CrI: 1.12, 4.34) and 2.64 times early in the morning (95%CrI: 1.07, 6.51) compared with sleeping in their own houses. Latrine use for defecation at night was associated with an increase of 2.71 times (95%CrI: 1.24, 5.91) among individuals from higher SES households compared with those from lower SES households, and 4.08 times higher (95%CrI: 2.24, 7.43) among those who perceived walking to latrines as safe compared with those who perceived access as neither safe nor unsafe or unsafe (S4 Table and Fig 5). The sensitivity analysis indicated similar trends to the results above (S5 Table and S6 Table).

## Discussion

Our study found that most individuals defecated in latrines, but urinated in open fields, with latrine use varying by age and time of day. Latrine use was less common among young children for both urination and defecation and among adolescents for defecation, compared with adults. Nighttime latrine use was consistently the lowest across all age groups, likely due to safety concerns when walking to the latrine. Despite limited handwashing infrastructure, all age groups were conscientious about washing hands before eating.

In the present study, consistent use for defecation was 66% among children and 91% among adults, which was comparable to a previous report showing that 66% of children and 88% of adults used latrines in Kenya [23]. However, consistent use for urination decreased to less than 20% among children and adults. As previous studies have shown [20,24–26,37,47,59], individuals do not necessarily use outdoor latrines simply because they own one. Latrine ownership alone is therefore insufficient to eliminate open defecation.

Barriers to latrine use varied by time of day and type of latrine use. Nighttime safety concerns appear to be a critical barrier to latrine use, potentially reflecting issues related to age, gender-based violence, social norms, or lighting infrastructure [23,40–43,47]. The wider credible interval observed for this association likely reflects low variability in perceived safety and latrine use, suggesting a polarized pattern in which individuals who felt safe at night tended to use latrines, whereas those who did not perceive safety were more likely to use open fields. Simple, low-cost interventions, such as promoting the use of flashlights and constructing latrines closer to households, may improve perceived safety and encourage more consistent latrine use at night [60].

Other factors associated with latrine use included having a caretaker with a higher level of education, mud floors in the latrines, sleeping in kitchens or other household structures, and belonging to a household of middle SES. Caretaker education was consistently associated with latrine use for urination across all times of day. Higher educational attainment may improve knowledge-related sanitation practices [61] and motivate caretakers to encourage and train children to use latrines [62]. The mechanisms underlying the association with latrine floor material and sleeping location remained unclear. Latrines with mud floors were more likely to be used than those with cement or tiled floors. One possible explanation is that dirt may be more visible on cement or tiles floors than mud floors. Because clean latrines have been associated with latrine use [25,34,63,64], individuals may fear leaving visible dirt after using latrines with cement or tiled floors [65,66]. This concern may create pressure to maintain cleanliness, leading individuals to prefer latrines with mud floors.

Sleeping in kitchen or other household structures was associated with greater latrine use for defecation at night and in the early morning compared with sleeping in their houses. Other contextual factors, such as the number of people sleeping in a given location and the surrounding environment, may also influence decisions about latrine use. For instance, fewer individuals may sleep in kitchens or other household structures than in their houses. When many people wake up simultaneously, individuals may need to wait for access to the latrine [20,47], whereas fewer individuals sleeping in the same space could reduce such delays. People may also avoid open defecation near kitchen structures to prevent unpleasant smells and flies attracted to human waste. In addition, the association between SES and latrine use may partly reflect differences in house locations and surrounding environmental conditions across poverty levels [67]. Households from both low- and high-SES groups may provide environments that facilitate open urination, such as remote locations or compounds with greater privacy due to fencing or larger land areas. Consequently, during early morning hours when latrines are more likely to be crowded, no significant difference in latrine use was observed between low and high SES groups. In contrast, individuals from middle-SES households, who may experience greater social pressure, were more likely to use latrines for urination in the early morning compared with those from low-SES households. During the nighttime, individuals from high-SES households were more likely to use latrine for defecation than those from low-SES households. This pattern may reflect differences in latrine quality and safety, as higher-SES households are more likely to have latrines with improved features (e.g., lighting or more stable structures) [68], which may encourage latrine use at night. Further research is needed to better understand how floor material, sleeping location, and SES interact to shape latrine use behaviors.

Consistent with previous findings [47], age was an important factor associated with latrine use in the present study. Our descriptive study showed that the elderly used latrines as frequently as other adult groups, contrary to several studies reporting a preference for open defecation among the elderly due to long-standing habits or less concerns about being seen outdoors [23,37,59]. However, despite reporting feeling safe walking to the latrine at night, some elderly individuals used buckets for urination during nighttime hours, suggesting physical or accessibility barriers may influence latrine use. These findings underscore the importance of adapting sanitation environments to meet the needs of the elderly by maintaining clear paths from houses to the latrines, constructing additional latrines closer to households, and installing supportive features such as handrails or seating within latrines [41,69].

Children were less likely to use latrines than adults. In Kenya, school-aged children are introduced to hygiene and WASH-related topics through the school curriculum, including handwashing in Class 1 (6–7 years) and Class 2 (7–8 years), and disease risks related to WASH in Class 6 (11–12 years), Class 7 (12–13 years), and Class 8 (13–14 years) [70]. Nevertheless, our results showed that children aged 4–10 years were less likely than adults to use latrines for both urination and defecation, and adolescents aged 11–17 years were less likely to use latrines for defecation, even after adjusting for perceived safety and other covariates. These findings are consistent with previous studies documenting a gap between sanitation knowledge and actual practices among children and adolescents [70–72]. This gap may reflect limited integration of practical WASH skills into standardized school curricula [70], lower comprehension of WASH concepts among younger children [71], and the persistence of habits formed early in life [37]. Although behavior change is challenging, health education remains a key strategy for improving latrine use. Community-based WASH-related health promotion has been shown to improve hygiene and sanitation behaviors [73]. Additionally, school children can serve as agents of change by sharing health messages or demonstrating positive behaviors within households and communities [74–76], even though gaps between knowledge and practice remain a concern. Exposure to a secondary education, learning from neighbors, and observing latrine use among peers have also been associated with increased latrine use [25,61,64,77,78].

In the present study, handwashing practices included both washing with water only and washing with soap. Although soap may not always be used, handwashing with water alone has been shown to reduce bacteria of potential fecal origin and decrease diarrheal diseases [45,79]. About 50% of individuals always practiced handwashing after defecation; however, few latrines were equipped with handwashing stations. Although people may wash their hands using water typically stored inside houses in rural Kenya [80,81], further research is needed to understand where and how handwashing occurs and whether soap is used.

### Limitations

First, we asked about individuals' general behaviors rather than behaviors during a specific period (e.g., the 48 hours prior to the date of the survey [47]). As a result, responses may be subject to reporting bias and non-differential misclassification, as self-reported behaviors may not accurately reflect their actual practices [47]. Second, the present study was a cross-sectional study conducted among households with an outdoor latrine. Hygiene behaviors may be different in other settings, such as schools or workplaces, and may vary by seasons [47]. Therefore, the findings may not represent behaviors across all contexts or throughout the year. Third, the self-reported data may be influenced by social desirability bias. Some respondents may have overreported latrine use and handwashing practices to present themselves favorably, whereas others may have underreported these behaviors due to expectations of future latrine or handwashing infrastructure support. These biases may have led to either overestimation or underestimation of actual hygiene practices. Finally, households with changes in family composition or latrine characteristics were included in the present study. While this approach enhanced external validity, such changes may have introduced misclassification of exposed variables. However, this potential bias is likely limited because latrine use behaviors were recorded at the individual level at the time of data collection. Moreover, sensitivity analyses excluding individuals from households that did not meet the eligibility criteria at the recruitment period showed the trends similar to the main findings.

## Conclusions

Our findings indicate that most individuals defecated in latrines but urinated in open fields across all time points. Compared with adults, young children were less likely to use latrines for both urination and defecation, and adolescents were less likely to use latrines for defecation. Additionally, perceived safety was associated with latrine use at night. Therefore, simple, low-cost interventions, such as promoting the use of flashlights, constructing latrines closer to households, and better connecting sanitation knowledge to daily practices, are crucial for improving sanitation behaviors.

## Supporting information

S1 Table Latrine use for urination and defecation during the day time, at night, and in the early morning by age and sex (n = 528). (DOCX)

S2 Table Handwashing practices after urination and defecation during the day time, at night, early morning, and before eating, by age and sex (n = 528). (DOCX)

S3 Table Unadjusted and adjusted analyses of factors associated with latrine use for urination during the daytime, at night, and in the early morning (n = 528). (DOCX)

S4 Table Unadjusted and adjusted analyses of factors associated with latrine use for defecation during the daytime, at night, and in the early morning (n = 528). (DOCX)

S5 Table Sensitivity analyses on factors associated with latrine use for urination using data set that met the criteria (n = 407). (DOCX)

S6 Table Sensitivity analyses on factors associated with latrine use for defecation using data set that met the criteria (n = 407). (DOCX)
