# Supplementary material for: Behavioral patterns in latrine use and handwashing in rural western Kenya: Age, time of day, and the role of perceived safety
Source: PLoS One. 2026 Mar 27;21(3):e0345954. doi: 10.1371/journal.pone.0345954 (PMC13028548; doi:10.1371/journal.pone.0345954)
Supplement: S1 Table — (DOCX) [file pone.0345954.s001.docx]

**S1 Table. Latrine use for urination and defecation during the day time, at night, and in the early morning by age and sex (n=528).**

| **Type** | **Variable** | **Age,** years, n (%) | | | | | **Children** | **Adults** |  |  | **All** |
| --- | --- | --- | --- | --- | --- | --- | --- | --- | --- | --- | --- |
|  |  | **4-10** | **11-17** | **18-39** | **40-59** | **60+** | **4-17** | **18+** | **Male** | **Female** |  |
| **Urination** | ***Daytime*** |  |  |  |  |  |  |  |  |  |  |
|  | Bucket | 9 (6.7) | 1 (0.8) | 1 (0.7) | 0 | 0 | 10 (3.8) | 1 (0.4) | 3 (1.3) | 8 (2.6) | 11 (2.1) |
|  | Open field | 110 (81.5) | 84 (65.6) | 98 (67.1) | 62 (72.1) | 23 (69.7) | 194 (73.8) | 183 (69.1) | 164 (72.6) | 213 (70.5) | 377 (71.4) |
|  | Latrine | 16 (11.9) | 43 (33.6) | 47 (32.2) | 24 (27.9) | 10 (30.3) | 59 (22.4) | 81 (30.6) | 59 (26.1) | 81 (26.8) | 140 (26.5) |
|  | ***Night*** |  |  |  |  |  |  |  |  |  |  |
|  | Bucket | 34 (25.2) | 15 (11.7) | 13 (8.9) | 3 (3.5) | 8 (24.2) | 49 (18.6) | 24 (9.1) | 20 (8.8) | 53 (17.5) | 73 (13.8) |
|  | Open field | 93 (68.9) | 85 (66.4) | 96 (65.8) | 63 (73.3) | 21 (63.6) | 178 (67.7) | 180 (67.9) | 161 (71.2) | 197 (65.2) | 358 (67.8) |
|  | Latrine | 8 (5.9) | 28 (21.9) | 37 (25.3) | 20 (23.3) | 4 (12.1) | 36 (13.7) | 61 (23.0) | 45 (19.9) | 52 (17.2) | 97 (18.4) |
|  | ***Early*** ***morning*** |  |  |  |  |  |  |  |  |  |  |
|  | Bucket | 8 (5.9) | 0 | 1 (0.7) | 0 | 0 | 8 (3.0) | 1 (0.4) | 4 (1.8) | 5 (1.7) | 9 (1.7) |
|  | Open field | 117 (86.7) | 98 (76.6) | 97 (66.4) | 63 (73.3) | 25 (75.8) | 215 (81.7) | 185 (69.8) | 173 (76.5) | 227 (75.2) | 400 (75.8) |
|  | Latrine | 10 (7.4) | 30 (23.4) | 48 (32.9) | 23 (26.7) | 8 (24.2) | 40 (15.2) | 79 (29.8) | 49 (21.7) | 70 (23.2) | 119 (22.5) |
| **Defecation** | ***Daytime*** |  |  |  |  |  |  |  |  |  |  |
|  | Bucket | 8 (5.9) | 1 (0.8) | 1 (0.7) | 0 | 0 | 9 (3.4) | 1 (0.4) | 4 (1.8) | 6 (2.0) | 10 (1.9) |
|  | Open field | 26 (19.3) | 6 (4.7) | 7 (4.8) | 2 (2.3) | 2 (6.1) | 32 (12.2) | 11 (4.2) | 17 (7.5) | 26 (8.6) | 43 (8.1) |
|  | Latrine | 101 (74.8) | 121 (94.5) | 138 (94.5) | 84 (97.7) | 31 (93.9) | 222 (84.4) | 253 (95.5) | 205 (90.7) | 270 (89.4) | 475 (90.0) |
|  | ***Night*** |  |  |  |  |  |  |  |  |  |  |
|  | Bucket | 24 (17.8) | 2 (1.6) | 3 (2.1) | 0 | 1 (3.0) | 26 (9.9) | 4 (1.5) | 9 (4.0) | 21 (7.0) | 30 (5.7) |
|  | Open field | 35 (25.9) | 17 (13.3) | 6 (4.1) | 1 (1.2) | 2 (6.1) | 52 (19.8) | 9 (3.4) | 20 (8.8) | 41 (13.6) | 61 (11.6) |
|  | Latrine | 76 (56.3) | 109 (85.2) | 137 (93.8) | 85 (98.8) | 30 (90.9) | 185 (70.3) | 252 (95.1) | 197 (87.2) | 240 (79.5) | 437 (82.8) |
|  | ***Early*** ***morning*** |  |  |  |  |  |  |  |  |  |  |
|  | Bucket | 7 (5.2) | 0 | 0 | 0 | 0 | 7 (2.7) | 0 | 4 (1.8) | 3 (1.0) | 7 (1.3) |
|  | Open field | 28 (20.7) | 7 (5.5) | 2 (1.4) | 2 (2.3) | 1 (3.0) | 35 (13.3) | 5 (1.9) | 16 (7.1) | 24 (7.9) | 40 (7.6) |
|  | Latrine | 100 (74.1) | 121 (94.5) | 144 (98.6) | 84 (97.7) | 32 (97.0) | 221 (84.0) | 260 (98.1) | 206 (91.2) | 275 (91.1) | 481 (91.1) |
| ***Total*** | | 135 (25.6) | 128 (24.2) | 146 (27.7) | 86 (16.3) | 33 (6.3) | 263 (49.8) | 265 (50.2) | 226 (42.8) | 302 (57.2) | 528 (100) |

^a^ Based on individual perception rather than quantitative assessment
