# Supplementary material for: Behavioral patterns in latrine use and handwashing in rural western Kenya: Age, time of day, and the role of perceived safety
Source: PLoS One. 2026 Mar 27;21(3):e0345954. doi: 10.1371/journal.pone.0345954 (PMC13028548; doi:10.1371/journal.pone.0345954)
Supplement: S2 Table — (DOCX) [file pone.0345954.s002.docx]

**S2 Table. Handwashing practices after urination and defecation during the day time, at night, in the early morning, and before eating, by age and sex (n=528).**

| **Type** | **Variable** | **Age,** years, n(%) | | | | | **Children** | **Adults** |  |  | **All** |
| --- | --- | --- | --- | --- | --- | --- | --- | --- | --- | --- | --- |
|  |  | **4-10** | **11-17** | **18-39** | **40-59** | **60+** | **4-17** | **18+** | **Male** | **Female** |  |
| ***Urination*** | ***Daytime*** |  |  |  |  |  |  |  |  |  |  |
|  | Never | 18 (13.3) | 10 (7.8) | 8 (5.5) | 4 (4.7) | 4 (12.1) | 28 (10.6) | 16 (6.0) | 20 (8.8) | 24 (7.9) | 44 (8.3) |
|  | Rarely | 14 (10.4) | 7 (5.5) | 10 (6.8) | 3 (3.5) | 2 (6.1) | 21 (8.0) | 15 (5.7) | 17 (7.5) | 19 (6.3) | 36 (6.8) |
|  | Sometimes | 75 (55.6) | 73 (57.0) | 73 (50.0) | 48 (55.8) | 18 (54.5) | 148 (56.3) | 139 (52.5) | 135 (59.7) | 152 (50.3) | 287 (54.4) |
|  | Always | 28 (20.7) | 38 (29.7) | 55 (37.7) | 31 (36.0) | 9 (27.3) | 66 (25.1) | 95 (35.8) | 54 (23.9) | 107 (35.4) | 161 (30.5) |
|  | ***Night*** |  |  |  |  |  |  |  |  |  |  |
|  | Never | 37 (27.4) | 43 (33.6) | 35 (24.0) | 17 (19.8) | 13 (39.4) | 80 (30.4) | 65 (24.5) | 59 (26.1) | 86 (28.5) | 145 (27.5) |
|  | Rarely | 14 (10.4) | 9 (7.0) | 10 (6.8) | 7 (8.1) | 4 (12.1) | 23 (8.7) | 21 (7.9) | 21 (9.3) | 23 (7.6) | 44 (8.3) |
|  | Sometimes | 59 (43.7) | 45 (35.2) | 53 (36.3) | 36 (41.9) | 10 (30.3) | 104 (39.5) | 99 (37.4) | 103 (45.6) | 100 (33.1) | 203 (38.4) |
|  | Always | 25 (18.5) | 31 (24.2) | 48 (32.9) | 26 (30.2) | 6 (18.2) | 56 (21.3) | 80 (30.2) | 43 (19.0) | 93 (30.8) | 136 (25.8) |
|  | ***Early*** ***morning*** |  |  |  |  |  |  |  |  |  |  |
|  | Never | 21 (15.6) | 18 (14.1) | 9 (6.2) | 4 (4.7) | 6 (18.2) | 39 (14.8) | 19 (7.2) | 22 (9.7) | 36 (11.9) | 58 (11.0) |
|  | Rarely | 14 (10.4) | 9 (7.0) | 13 (8.9) | 11 (12.8) | 2 (6.1) | 23 (8.7) | 26 (9.8) | 28 (12.4) | 21 (7.0) | 49 (9.3) |
|  | Sometimes | 69 (51.1) | 53 (41.4) | 65 (44.5) | 36 (41.9) | 14 (42.4) | 122 (46.4) | 115 (43.4) | 115 (50.9) | 122 (40.4) | 237 (44.9) |
|  | Always | 31 (23.0) | 48 (37.5) | 59 (40.4) | 35 (40.7) | 11 (33.3) | 79 (30.0) | 105 (39.6) | 61 (27.0) | 123 (40.7) | 184 (34.8) |
| ***Defecation*** | ***Daytime*** |  |  |  |  |  |  |  |  |  |  |
|  | Never | 8 (5.9) | 4 (3.1) | 4 (2.7) | 0 | 0 | 12 (4.6) | 4 (1.5) | 7 (3.1) | 9 (3.0) | 16 (3.0) |
|  | Rarely | 9 (6.7) | 3 (2.3) | 8 (5.5) | 4 (4.7) | 1 (3.0) | 12 (4.6) | 13 (4.9) | 11 (4.9) | 14 (4.6) | 25 (4.7) |
|  | Sometimes | 67 (49.6) | 65 (50.8) | 57 (39.0) | 36 (41.9) | 15 (45.5) | 132 (50.2) | 108 (40.8) | 110 (48.7) | 130 (43.0) | 240 (45.5) |
|  | Always | 51 (37.8) | 56 (43.8) | 77 (52.7) | 46 (53.5) | 17 (51.5) | 107 (40.7) | 140 (52.8) | 98 (43.4) | 149 (49.3) | 247 (46.8) |
|  | ***Night*** |  |  |  |  |  |  |  |  |  |  |
|  | Never | 33 (24.4) | 29 (22.7) | 30 (20.5) | 13 (15.1) | 9 (27.3) | 62 (23.6) | 52 (19.6) | 41 (18.1) | 73 (24.2) | 114 (21.6) |
|  | Rarely | 10 (7.4) | 8 (6.2) | 9 (6.2) | 5 (5.8) | 3 (9.1) | 18 (6.8) | 17 (6.4) | 16 (7.1) | 19 (6.3) | 35 (6.6) |
|  | Sometimes | 52 (38.5) | 44 (34.4) | 39 (26.7) | 29 (33.7) | 6 (18.2) | 96 (36.5) | 74 (27.9) | 86 (38.1) | 84 (27.8) | 170 (32.2) |
|  | Always | 40 (29.6) | 47 (36.7) | 68 (46.6) | 39 (45.3) | 15 (45.5) | 87 (33.1) | 122 (46.0) | 83 (36.7) | 126 (41.7) | 209 (39.6) |
|  | ***Early*** ***morning*** |  |  |  |  |  |  |  |  |  |  |
|  | Never | 9 (6.7) | 5 (3.9) | 6 (4.1) | 0 | 2 (6.1) | 14 (5.3) | 8 (3.0) | 11 (4.9) | 11 (3.6) | 22 (4.2) |
|  | Rarely | 12 (8.9) | 9 (7.0) | 11 (7.5) | 4 (4.7) | 1 (3.0) | 21 (8.0) | 16 (6.0) | 17 (7.5) | 20 (6.6) | 37 (7.0) |
|  | Sometimes | 65 (48.1) | 51 (39.8) | 44 (30.1) | 36 (41.9) | 11 (33.3) | 116 (44.1) | 91 (34.3) | 95 (42.0) | 112 (37.1) | 207 (39.2) |
|  | Always | 49 (36.3) | 63 (49.2) | 85 (58.2) | 46 (53.5) | 19 (57.6) | 112 (42.6) | 150 (56.6) | 103 (45.6) | 159 (52.6) | 262 (49.6) |
| **Before eating** | ***Before eating*** |  |  |  |  |  |  |  |  |  |  |
|  | Never | 0 | 0 | 1 (0.7) | 0 | 0 | 0 | 1 (0.4) | 0 | 1 (0.3) | 1 (0.2) |
|  | Rarely | 2 (1.5) | 0 | 0 | 0 | 0 | 2 (0.8) | 0 | 0 | 2 (0.7) | 2 (0.4) |
|  | Sometimes | 0 | 2 (1.6) | 2 (1.4) | 1 (1.2) | 0 | 2 (0.8) | 3 (1.1) | 2 (0.9) | 3 (1.0) | 5 (0.9) |
|  | Always | 133 (98.5) | 126 (98.4) | 143 (97.9) | 85 (98.8) | 33 (100) | 259 (98.5) | 261 (98.5) | 224 (99.1) | 296 (98.0) | 520 (98.5) |
| ***Total*** | | 135 (25.6) | 128 (24.2) | 146 (27.7) | 86 (16.3) | 33 (6.3) | 263 (49.8) | 265 (50.2) | 226 (42.8) | 302 (57.2) | 528 (100) |
