# Supplementary material for: Behavioral patterns in latrine use and handwashing in rural western Kenya: Age, time of day, and the role of perceived safety
Source: PLoS One. 2026 Mar 27;21(3):e0345954. doi: 10.1371/journal.pone.0345954 (PMC13028548; doi:10.1371/journal.pone.0345954)
Supplement: S3 Table — (DOCX) [file pone.0345954.s003.docx]

**S3 Table.** **Unadjusted and adjusted analyses of factors associated with latrine use for urination during the daytime, at night, and in the early morning (n=528).**

| *Predictors* | **Daytime** | | | **Night** | | **Early morning** | |
| --- | --- | --- | --- | --- | --- | --- | --- |
|  | PR (95%CrI) | aPR (95%CrI) | | PR (95%CrI) | aPR (95%CrI) | PR (95%CrI) | aPR (95%CrI) |
| ***Sex*** *(ref.*  Male*)* |  | |  |  |  |  |  |
| Female | 1.04 (0.70,1.54) | | 0.99 (0.65,1.50) | 0.84 (0.54,1.30) | 1.06 (0.65,1.74) | 1.09 (0.72,1.65) | 1.02 (0.65,1.60) |
| ***Age,*** *year (ref.*18-39*)* |  | |  |  |  |  |  |
| 4-10 | 0.28* (0.15,0.52) | | 0.28* (0.15,0.53) | 0.18* (0.08,0.40) | 0.41* (0.17,0.98) | 0.16* (0.08,0.33) | 0.15* (0.07,0.32) |
| 11-17 | 1.07 (0.64,1.77) | | 1.23 (0.72,2.10) | 0.82 (0.47,1.44) | 1.11 (0.60,2.06) | 0.62 (0.36,1.06) | 0.69 (0.39,1.22) |
| 40-59 | 0.81 (0.45,1.45) | | 0.77 (0.42,1.42) | 0.89 (0.47,1.65) | 0.92 (0.47,1.78) | 0.74 (0.41,1.33) | 0.77 (0.41,1.44) |
| 60+ | 0.90 (0.39,2.03) | | 0.94 (0.39,2.25) | 0.37 (0.12,1.12) | 0.40 (0.12,1.28) | 0.63 (0.26,1.50) | 0.67 (0.26,1.70) |
| ***Education level of caretaker*** *(ref.* Incomplete primary*)* |  | |  |  |  |  |  |
| Completed primary | 1.26 (0.76,2.08) | | 1.49 (0.87,2.56) | 1.12 (0.63,2.01) | 1.20 (0.64,2.26) | 1.29 (0.76,2.21) | 1.36 (0.76,2.44) |
| Completed secondary | 2.45* (1.45,4.14) | | 2.41* (1.34,4.36) | 2.41* (1.34,4.35) | 2.64* (1.35,5.15) | 2.40* (1.38,4.17) | 2.16* (1.15,4.06) |
| ***SES*** *(ref.*  Low*)* |  | |  |  |  |  |  |
| Middle | 1.47 (0.90,2.42) | | 1.57 (0.92,2.67) | 1.63 (0.92,2.89) | 1.78 (0.95,3.35) | 1.74* (1.03,2.93) | 2.05* (1.16,3.62) |
| High | 1.34 (0.81,2.20) | | 1.43 (0.81,2.52) | 1.41 (0.79,2.52) | 1.43 (0.73,2.79) | 1.21 (0.70,2.06) | 1.39 (0.75,2.57) |
| ***Num of individuals potentially using latrines*** | 0.95* (0.90,0.99) | | 0.97 (0.92,1.02) | 0.99 (0.94,1.03) | 1.01 (0.95,1.07) | 0.97 (0.92,1.01) | 1.00 (0.95,1.05) |
| ***Type of latrines*** *(ref.* Pit*)* |  | |  |  |  |  |  |
| VIP | 1.67 (0.93,3.00) | | 1.46 (0.70,3.03) | 0.82 (0.39,1.74) | 0.70 (0.28,1.78) | 1.61 (0.87,2.97) | 1.85 (0.83,4.11) |
| ***Floor in latrine*** *(ref.* Cement/tiles*)* |  | |  |  |  |  |  |
| Mud/other | 1.16 (0.78,1.72) | | 1.79* (1.07,2.99) | 1.32 (0.84,2.10) | 1.61 (0.90,2.88) | 1.39 (0.91,2.12) | 2.22* (1.26,3.88) |
| ***Feces around latrines*** *(ref.* Yes*)* |  | |  |  |  |  |  |
| No | 1.89* (1.15,3.09) | | 1.66 (0.97,2.85) | 1.61 (0.92,2.80) | 1.54  (0.83, 2.85) | 1.77* (1.05,2.97) | 1.56 (0.87,2.78) |
| ***Distance from houses to* *latrines,*** *m* | 0.99 (0.98,1.01) | | 0.99 (0.97,1.01) | - | - | - | - |
| ***Sleeping place*** *(ref*. Own house *)* |  | |  |  |  |  |  |
| Kitchen/other | - | | - | 1.21 (0.76,1.94) | 1.03  (0.59, 1.77) | 1.34 (0.87,2.07) | 1.07 (0.65,1.76) |
| ***Safety walking to a latrine*** *(ref.* Neither/unsafe*)* |  | |  |  |  |  |  |
| Safe | - | | - | 11.40* (4.10,31.67) | 9.47* (3.17,28.28) | - | - |
| ***Distance from sleeping places to latrines*** | - | | - | 1.00 (0.98,1.02) | 0.99 (0.97,1.01) | 0.99 (0.97,1.01) | 0.98 (0.96,1.00) |

*Credible evidence

aPR, adjusted prevalence ratio; CrI, credible interval; num, number; PR, prevalence ratio; *ref*, reference; SES, socio economic status; VIP, ventilated improved pit
