# Supplementary material for: Behavioral patterns in latrine use and handwashing in rural western Kenya: Age, time of day, and the role of perceived safety
Source: PLoS One. 2026 Mar 27;21(3):e0345954. doi: 10.1371/journal.pone.0345954 (PMC13028548; doi:10.1371/journal.pone.0345954)
Supplement: S4 Table — (DOCX) [file pone.0345954.s004.docx]

**S4 Table. Unadjusted and adjusted analyses of factors associated with latrine use for defecation during the daytime, at night, and in the early morning (n=528).**

| *Predictors* | **Daytime** | | | **Night** | | **Early morning** | |
| --- | --- | --- | --- | --- | --- | --- | --- |
|  | PR (95%CrI) | aPR (95%CrI) | | PR (95%CrI) | aPR (95%CrI) | PR (95%CrI) | aPR (95%CrI) |
| ***Sex*** *(ref.* Male*)* |  | |  |  |  |  |  |
| Female | 0.86 (0.48,1.53) | | 0.78 (0.41,1.46) | 0.56* (0.35,0.91) | 0.55 (0.30,1.01) | 0.98 (0.54,1.80) | 0.91 (0.46,1.79) |
| ***Age,*** *year (ref.* 18+*)* |  | |  |  |  |  |  |
| 4-10 | 0.14* (0.07,0.27) | | 0.12* (0.06,0.25) | 0.06* (0.03,0.12) | 0.08* (0.04,0.18) | 0.05* (0.02,0.13) | 0.04* (0.02,0.11) |
| 11-17 | 0.84 (0.32,2.19) | | 0.77 (0.29,2.02) | 0.29* (0.14,0.61) | 0.23* (0.10,0.53) | 0.32 (0.10,1.04) | 0.25* (0.08,0.84) |
| ***Education level of caretaker****(ref.* Incomplete primary*)* |  | |  |  |  |  |  |
| Completed primary | 0.57 (0.27,1.18) | | 0.74 (0.34,1.63) | 0.84 (0.49,1.46) | 1.26 (0.65, 2.45) | 0.95 (0.46,1.97) | 1.31 (0.58,2.98) |
| Completed secondary | 0.72 (0.32,1.65) | | 0.77 (0.30,1.96) | 0.94 (0.51,1.75) | 0.90 (0.40,2.00) | 0.95 (0.42,2.12) | 0.80 (0.30,2.11) |
| ***SES*** *(ref.*  Low*)* |  | |  |  |  |  |  |
| Middle | 1.09 (0.56,2.11) | | 1.04 (0.49,2.21) | 1.30 (0.76,2.21) | 1.61 (0.82,3.17) | 1.14 (0.57,2.30) | 1.11 (0.49,2.51) |
| High | 1.94 (0.93,4.07) | | 1.75 (0.72,4.25) | 2.27* (1.27,4.06) | 2.71* (1.24,5.91) | 1.86 (0.86,4.04) | 2.08 (0.80,5.42) |
| ***Num of individuals potentially using latrines*** | 0.95 (0.90,1.00) | | 0.95 (0.89,1.02) | 0.95* (0.91,0.99) | 0.95 (0.90,1.01) | 0.98 (0.92,1.03) | 0.99 (0.92,1.07) |
| ***Type of latrines*** *(ref.* Pit*)* |  | |  |  |  |  |  |
| VIP | 1.22 (0.46,3.21) | | 1.05 (0.32,3.49) | 1.56 (0.68,3.58) | 1.82 (0.59,5.60) | 1.41 (0.49,4.10) | 1.54 (0.41,5.76) |
| ***Floor in latrines*** *(ref.* Cement/tiles*)* |  | |  |  |  |  |  |
| Mud/other | 0.88 (0.49,1.58) | | 0.96 (0.45,2.06) | 1.01 (0.64,1.60) | 1.14 (0.58,2.24) | 1.10 (0.60,2.02) | 1.39 (0.61,3.14) |
| ***Feces around latrines*** *(ref.* Yes*)* |  | |  |  |  |  |  |
| No | 0.84 (0.43,1.66) | | 0.75 (0.34,1.65) | 0.87 (0.51,1.48) | 0.83 (0.42,1.63) | 1.01 (0.51,2.00) | 0.99 (0.44,2.27) |
| ***Distance from house to* *latrine,*** *m* | 0.99 (0.97,1.01) | | 0.99 (0.97,1.02) | - | - | - | - |
| ***Sleeping place*** *(ref*. Own house*)* |  | |  |  |  |  |  |
| Kitchen/other | - | | - | 1.91* (1.01,3.32) | 2.21* (1.12,4.34) | 2.71* (1.19,6.20) | 2.64* (1.07,6.51) |
| ***Safety walking to a latrine*** *(ref.* Neither/unsafe*)* |  | |  |  |  |  |  |
| Safe | - | | - | 8.87* (5.40,14.57) | 4.08* (2.24,7.43) | - | - |
| ***Distance from sleeping place to latrine*** | - | | - | 0.99 (0.97,1.0) | 0.98 (0.96,1.01) | 1.00 (0.98,1.03) | 1.00 (0.97,1.03) |

*Credible evidence

aPR, adjusted prevalence ratio; CrI, credible interval; num, number; PR, prevalence ratio; *ref*, reference; SES, socio economic status; VIP, ventilated improved pit
