# Supplementary material for: Behavioral patterns in latrine use and handwashing in rural western Kenya: Age, time of day, and the role of perceived safety
Source: PLoS One. 2026 Mar 27;21(3):e0345954. doi: 10.1371/journal.pone.0345954 (PMC13028548; doi:10.1371/journal.pone.0345954)
Supplement: S5 Table — (DOCX) [file pone.0345954.s005.docx]

**S5 Table**. **Sensitivity analyses on factors associated with latrine use for urination using dataset that met the criteria (n=407).**

| *Predictors* | **Daytime** | | | **Night** | | **Early morning** | |
| --- | --- | --- | --- | --- | --- | --- | --- |
|  | PR (95%CrI) | aPR (95%CrI) | | PR (95%CrI) | aPR (95%CrI) | PR (95%CrI) | aPR (95%CrI) |
| ***Sex*** *(ref.*  Male*)* |  | |  |  |  |  |  |
| Female | 1.2 (0.77,1.88) | | 1.06 (0.64,1.73 | 0.94 (0.56,1.57) | 0.96 (0.54,1.72) | 1.26 (0.78,2.05) | 1.09 (0.63,1.87) |
| ***Age,*** *year (ref.*18-39*)* |  | |  |  |  |  |  |
| 4-10 | 0.17* (0.08,0.38) | | 0.15* (0.07,0.35) | 0.04* (0.01,0.19) | 0.07* (0.01,0.32) | 0.07* (0.02,0.20) | 0.06* (0.02,0.18) |
| 11-17 | 1.07 (0.61,1.89) | | 1.06 (0.58,1.95) | 0.82 (0.44,1.55) | 0.84 (0.41,1.69) | 0.61 (0.33,1.11) | 0.53 (0.27,1.02) |
| 40-59 | 0.88 (0.45,1.73) | | 0.73 (0.36,1.48) | 0.96 (0.47,1.98) | 0.86 (0.39,1.88) | 0.76 (0.38,1.51) | 0.75 (0.35,1.57) |
| 60+ | 1.44 (0.53,3.93) | | 1.12 (0.38,3.28) | 0.49 (0.13,1.84) | 0.36 (0.09,1.44) | 0.68 (0.22,2.04) | 0.54 (0.17,1.78) |
| ***Education level of caretaker*** *(ref.* Incomplete primary*)* |  | |  |  |  |  |  |
| Completed primary | 1.02 (0.59,1.79) | | 1.36 (0.73,2.54) | 0.85 (0.44,1.64) | 0.97 (0.47,2.01) | 1.06 (0.57,1.96) | 1.19 (0.59,2.37) |
| Completed secondary | 1.89* (1.04,3.42) | | 1.97* (1.00,3.89) | 1.93 (0.93,3.59) | 2.00 (0.93,4.31) | 2.01* (1.06,3.82) | 1.91 (0.91,4.01) |
| ***SES*** *(ref.*  Low*)* |  | |  |  |  |  |  |
| Middle | 0.90 (0.50,1.63) | | 0.93 (0.49,1.77) | 0.91 (0.45,1.84) | 0.97 (0.45,2.12) | 1.17 (0.62,2.22) | 1.28 (0.63,2.60) |
| High | 1.05 (0.60,1.84) | | 1.31 (0.69,2.50) | 1.21 (0.62,2.33) | 1.33 (0.62,2.85) | 1.08 (0.58,2.02) | 1.34 (0.65,2.76) |
| ***Num of individuals potentially using latrines*** | 0.91* (0.85,0.96) | | 0.91 (0.85,0.98) | 0.95 (0.90,1.01) | 0.97 (0.90,1.04) | 0.94 (0.89,1.00) | 0.97 (0.90,1.04) |
| ***Type of latrines*** *(ref.* Pit*)* |  | |  |  |  |  |  |
| VIP | 1.67 (0.89,3.12) | | 1.47 (0.66,3.25) | 0.88 (0.39,1.96) | 0.62 (0.23,1.68) | 1.54 (0.79,3.01) | 1.31 (0.54,3.15) |
| ***Floor in latrine*** *(ref.* Cement/tiles*)* |  | |  |  |  |  |  |
| Mud/other | 1.24 (0.80,1.93) | | 1.84* (1.05,3.24) | 1.15 (0.69,1.93) | 1.33 (0.70,2.52) | 1.34 (0.83,2.16) | 1.85* (1.05,3.62) |
| ***Feces around latrines*** *(ref.* Yes*)* |  | |  |  |  |  |  |
| No | 2.25* (1.25,4.06) | | 2.00 (1.04,3.83) | 2.21* (1.09,4.51) | 2.19* (1.01,4.78) | 2.63* (1.33,5.18) | 2.53* (1.20,5.32) |
| ***Distance from houses to* *latrines,*** *m* | 0.99 (0.97,1.01) | | 0.99 (0.97,1.01) | - | - | - | - |
| ***Sleeping place*** *(ref*. Own house *)* |  | |  |  |  |  |  |
| Kitchen/other | - | | - | 1.36 (0.79,2.32) | 1.25 (0.65,2.42) | 1.59 (0.97,2.60) | 1.48 (0.81,2.71) |
| ***Safety walking to a latrine*** *(ref.* Neither/unsafe*)* |  | |  |  |  |  |  |
| Safe | - | | - | 8.53* (3.03,24.00) | 4.21* (1.36,13.02) | - | - |
| ***Distance from sleeping places to latrines,*** *m* | - | | - | 0.99 (0.97,1.01) | 1.00 (0.97,1.02) | 0.99 (0.97,1.00) | 0.98 (0.96,1.01) |

*Credible evidence

aPR, adjusted prevalence ratio; CrI, credible interval; num, number; PR, prevalence ratio; *ref*, reference; SES, socio economic status; VIP, ventilated improved pit
