# Supplementary material for: Behavioral patterns in latrine use and handwashing in rural western Kenya: Age, time of day, and the role of perceived safety
Source: PLoS One. 2026 Mar 27;21(3):e0345954. doi: 10.1371/journal.pone.0345954 (PMC13028548; doi:10.1371/journal.pone.0345954)
Supplement: S6 Table — (DOCX) [file pone.0345954.s006.docx]

**S6 Table**. **Sensitivity analyses on factors associated with latrine use for defecation using dataset that met the criteria (n=407).**

| *Predictors* | **Daytime** | | **Night** | | **Early morning** | |
| --- | --- | --- | --- | --- | --- | --- |
|  | PR (95%CrI) | aPR (95%CrI) | PR (95%CrI) | aPR (95%CrI) | PR (95%CrI) | aPR (95%CrI) |
| ***Sex*** *(ref.*  Male*)* |  |  |  |  |  |  |
| Female | 0.87 (0.46,1.67) | 0.78 (0.38,1.62) | 0.52* (0.30,0.91) | 0.47* (0.24,0.92) | 1.17 (0.60,2.32) | 1.27 (0.58,2.78) |
| ***Age,*** *year (ref.*18+*)* |  |  |  |  |  |  |
| 4-10 | 0.10* (0.04,0.23) | 0.08* (0.03,0.20) | 0.05* (0.02,0.12) | 0.07* (0.02,0.17) | 0.02* (0.01,0.10) | 0.02* (0.00,0.08) |
| 11-17 | 0.73 (0.24,2.23) | 0.69 (0.22,2.17) | 0.22* (0.09,0.55) | 0.22* (0.08,0.59) | 0.17* (0.03,0.86) | 0.12* (0.02,0.64) |
| ***Education level of caretaker*** *(ref.* Incomplete primary*)* |  |  |  |  |  |  |
| Completed primary | 0.56 (0.24,1.31) | 0.71 (0.28,1.85) | 0.89 (0.47,1.68) | 1.22 (0.56,2.68) | 0.64 (0.26,1.59) | 0.66 (0.24,1.84) |
| Completed secondary | 0.65 (0.25,1.65) | 0.62 (0.20,1.85) | 0.85 (0.42,1.72) | 0.78 (0.31,1.93) | 0.56 (0.21,1.47) | 0.40 (0.12,1.29) |
| ***SES*** *(ref.*  Low*)* |  |  |  |  |  |  |
| Middle | 1.18 (0.56,2.51) | 1.06 (0.43,2.60) | 1.15 (0.61,2.18) | 1.50 (0.67,3.35) | 0.98 (0.42,2.29) | 0.84 (0.31,2.28) |
| High | 2.53* (1.10,5.83) | 2.59 (0.94,7.13) | 2.02*  (1.04,3.93) | 2.73* (1.14,6.51) | 1.54 (0.64,3.71) | 1.71 (0.58,5.03) |
| ***Num of individuals potentially using latrines*** | 0.93* (0.88,0.99) | 0.90* (0.84,0.97) | 0.95 (0.91,1.00) | 0.94 (0.89,1.01) | 0.97 (0.91,1.03) | 0.96 (0.88,1.05) |
| ***Type of latrines*** *(ref.* Pit*)* |  |  |  |  |  |  |
| VIP | 1.13 (0.42,3.02) | 0.96 (0.27,3.41) | 1.35 (0.68,3.58) | 1.94 (0.62,6.06) | 1.26 (0.43,3.72) | 1.84 (0.47,7.13) |
| ***Floor in latrine*** *(ref.* Cement/tiles*)* |  |  |  |  |  |  |
| Mud/other | 0.88 (0.46,1.65) | 0.83 (0.35,1.97) | 1.01 (0.64,1.60) | 1.36 (0.65,2.85) | 1.23 (0.63,2.43) | 1.31 (0.53,3.29) |
| ***Feces around latrines*** *(ref.* Yes*)* |  |  |  |  |  |  |
| No | 0.80 (0.37,1.73) | 0.70 (0.28,1.77) | 0.87 (0.51,1.48) | 1.09 (0.50,2.35) | 0.84 (0.37,1.90) | 0.69 (0.26,1.87) |
| ***Distance from houses to* *latrines,*** *m* | 0.99 (0.97,1.02) | 1.01 (0.98,1.04) | - | - | - | - |
| ***Sleeping place*** *(ref*. Own house *)* |  |  |  |  |  |  |
| Kitchen/other | - | - | 1.91* (1.01,3.32) | 1.53* (0.74,3.17) | 2.14 (0.91,5.01) | 2.32* (0.88,6.11) |
| ***Safety walking to a latrine*** *(ref.* Neither/unsafe*)* |  |  |  |  |  |  |
| Safe | - | - | 8.87* (5.40,14.57) | 3.02* (1.51,6.04) | - | - |
| ***Distance from sleeping places to latrines,*** *m* | - | - | 0.99 (0.97,1.0) | 0.99 (0.96,1.01) | 1.00 (0.98,1.03) | 1.01 (0.98,1.04) |

*Credible evidence

aPR, adjusted prevalence ratio; CrI, credible interval; num, number; PR, prevalence ratio; *ref*, reference; SES, socio economic status; VIP, ventilated improved pit
